# Trends in the management and outcomes of patients admitted with acute coronary syndrome complicated by cardiogenic shock over the past decade: Real world data from the acute coronary syndrome Israeli survey (ACSIS)

**DOI:** 10.18632/oncotarget.17152

**Published:** 2017-04-17

**Authors:** Eran Kalmanovich, Alex Blatt, Svetlana Brener, Meital Shlezinger, Nir Shlomo, Zvi Vered, Hanoch Hod, Ilan Goldenberg, Gabby Elbaz-Greener

**Affiliations:** ^1^ Department of Cardiology, Assaf Harofeh Medical Center, Sackler Faculty of Medicine, Tel Aviv University, Tel Aviv, Israel; ^2^ The Heart Center, Chaim Sheba Medical Center, Tel Hashomer, Sackler Faculty of Medicine, Tel Aviv University, Tel Aviv, Israel

**Keywords:** cardiogenic shock, acute coronary syndrome, acute coronary syndrome Israeli surveys (ACSIS)

## Abstract

Registries and other cohorts have demonstrated that early revascularization improve the survival of patients presenting with Cardiogenic Shock (CS) completing Aute coronary syndrome (ACS). Our aim was to describe the change in the clinical characteristics of these patients and their management and their outcome. The study population comprised 224 patients who were admitted with ACS complicated by cardiogenic shock who were enrolled in the prospective biannual Acute Coronary Syndrome Israeli Surveys (ACSIS) between 2000 and 2013 (1.7% of all patients admitted with ACS during the study period). Survey periods were categorized as early (years 2000-2004) and late (year 2006-2013).

The rate of cardiogenic shock complicated ACS declined from 1.8% between the years 2000-2004 to 1.5% during the years 2006-2013. The clinical presentation in both the early and late groups was similar. During the index hospitalization primary percutaneous coronary intervention (PPCI) was more frequently employed during the late surveys [31% vs. 58% (p<0.001)], while fibrinolysis therapy was not used in the late surveys group [27% vs. 0.0% (p=<0.001)]. Compared to patients enrolled in the early surveys, those enrolled in the late survey group experienced significantly lower mortality rates at 7-days (44% vs. 30%, respectively; p=0.03). However, this difference was no longer statistically significant at 30-days (52.8% vs. 46.4%, respectively, p=0.34) and 1-year (63% vs. 53.2%, respectively, p=0.14). Similarly, the rate of major adverse cardiac events (MACE) at 30-days was similar between the two groups (57.4% vs. 47.4%, respectively, p=0.13).

Our findings indicate that patients admitted with ACS complicated by cardiogenic shock still experience very high rates of MACE and mortality during follow-up, despite a significant increase in the use of PPCI in this population over the past decade.

## INTRODUCTION

According to different registries, cardiogenic shock (CS) complicates patients presenting with acute coronary syndromes (ACS) up to one tenth of cases. The mortality from CS remains high, in spite of a decline in overall mortality and advances in medical management of patients presenting with ACS [1–5].

CS is characterized clinically by low cardiac output syndrome; systemic hypotension and vital organ hypoperfusion. Most cases of CS result from left ventricular pump failure due to extensive damage to the myocardium. Secondary infract - related mechanical complications are responsible for the remaining causes of CS in the setting of ACS [6–7].

In recent years there has been a gradual decline in the mortality of patients presenting with CS. This decline is attributed mainly to the practice of early and urgent primary revascularization. In the past two decades accumulating evidence showed that early reperfusion leads to gradual decrease in mortality and rate of complications due to myocardial infarction [1–5]. The “Should We Emergently Revascularize Occluded Coronaries for Cardiogenic Shock (SHOCK) trial” was the first to demonstrate an improvement of long term survival of patients with acute myocardial infarction complicated by cardiogenic shock who were assigned for aggressive medical therapy and early revascularization compared to aggressive medical therapy alone [8, 9]. Both AHA/ACC and ECS guidelines recommend on emergency revascularization with either percutaneous coronary intervention or coronary artery bypass surgery in suitable patients. (Class I, evidence B) [10, 11].

Data from European and North-American registries report on a gradual decrease in mortality rates over the past decade. A recent large cohort study from the Nationwide Inpatient Sample (NIS) in the US examining the incidence, management, and outcome of cardiogenic shock complicating ST-Elevation myocardial infarction (STEMI), found that during the 8 years of observation there was an increase in the incidence of cardiogenic shock in patients hospitalized with STEMI in the United States, with a significant increase in early mechanical revascularization and intra-aortic balloon pump (IABP) use in these patients [3]. Similar report from Italian registries over 14 years showed that there are over time trends in both the clinical characteristics and management of patients with CS complication ACS [5]. Based on these data from those and other cohorts, it was shown that increased rate of intervention was associated with a parallel decline in in-hospital mortality [5]. In the present study we sought to examine the temporal trends in the incidence of CS, characteristics, treatment and in-hospital outcome in patients enrolled in the Acute Coronary Syndrome Israeli Surveys (ACSIS) performed between 2000-2013.

## RESULTS

A total of 13,434 ACS patients were enrolled in the ACSIS surveys between 2000-2013, among whom 224 were complicated by CS. The prevalence of patients admitted with cardiogenic shock was 1.8% in the early surveys years, and 1.5% in the late survey groups.

### Baseline characteristics

The baseline characteristics of the CS patients by the survey periods are described in Table 1. No statistically significant differences between the two groups were shown with regard to baseline clinical risk factors, including age, gender and past history of coronary artery disease. However, diabetes mellitus, hypertension, and dyslipidemia were more frequent among patients enrolled in more recent surveys. Cardiovascular medications were admitted at a similar frequency to the two groups, with the exception of anti-hyperglycemic and lipid lowering agents which were administered at a higher frequency to patients enrolled in the later surveys group.

**Table 1:** Demographic and clinical characteristics

	Early Surveys(2000-2004)N=108	Late Surveys(2006-2013)N=116	p value
Gender (female)	28.7%	31%	0.7
Median Age	70.52±13.67	68.95±13.22	0.35
Age above >75 years	47.22%	38.79%	0.2
**Cardiovascular risk factor and cardiovascular history**			
History of Diabetes	28%	41.7%	0.03
History of HTN	50.5%	64.9%	0.029
History of dyslipidemia	31.8%	63.6%	0.001
Family history of CAD	10.3%	18.1%	0.11
Mean BMI	26.43±4.49	27.68±5.29	0.23
History of MI	33.6%	30.1%	0.57
Past PCI	29.9%	7.8%	0.1
Past CABG	4.7%	21.2%	0.33
Past CAV/TIA	19.4%	8.8%	0.02
History of CHF	13.1%	16.8%	0.58
History of CRF	13.9%	14.9%	0.82
History of PVD	13.1%	10.6%	0.57
**Chronic Medication**			
ASA	52.5%	44.9%	0.34
Clopidogrel	1.6%	5.7%	0/19
BB	38.1%	39.8	0.82
ACEi	28.6%	38.1%	0.2
CCB	19.4%	18.9%	0.92
Nitrate	28.6%	9.4%	0.001
Lipid lowering agents	15.7%	37.1%	0.0003
Insulin	3.2%	4.5%	0.65
Anti-Hyperglycemic	15.9	29.5	0.04

### Clinical presentation and management

The clinical presentation in both the early and late groups was similar, with almost half of the patients in both groups presented with typical angina (Table 2), which often had lasted more than 24 hours prior to admission. Dyspnea or heart failure as the initial presentation was more prevalent in the late surveys group [29.6% vs. 43.1% (p=0.03)]. Approximately one third of patients in the late surveys group presented initially with syncope or sudden cardiac death compared to one tenth of patients in early surveys groups [33.6% vs. 12.0% (p<0.001)]. Arrhythmias were also more prevalent in the late surveys group.

**Table 2:** Clinical presentation

	Early Surveys (2000-2004)N=108	Late Surveys(2006-2013)N=116	p value
**Typical Angina**	49.1%	41.4%	0.24
**Angina > 24 hours before admission**	29.9%	20%	0.09
**Atypical Angina**	7.4%	13.6%	0.16
**Dyspnea or heart failure**	29.6%	43.1%	0.03
**Syncope or aborted SCD**	12%	33.6%	<0.001
**Arrhythmia**	9.3%	19.8%	0.025

**Figure 1:**
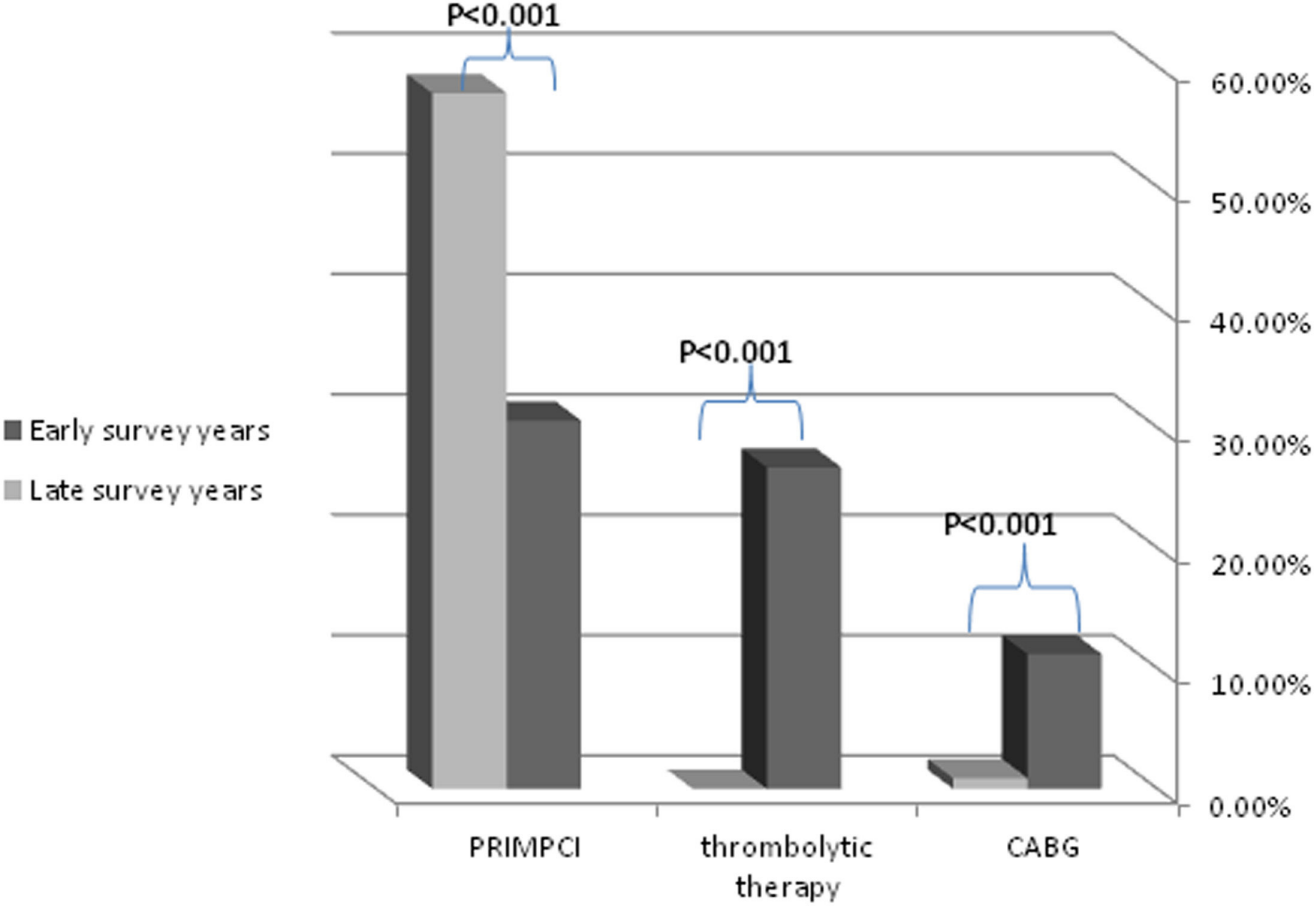
Trends in the frequency of revascularization methods

Patients from the late surveys group were more often admitted directly to intensive cardiac care unit than those in the early surveys groups. Both patient groups presented mainly with ST segment elevation in their initial ECG recording [72.45% vs. 75.25% (p=0.6)] most commonly anterior MI (43.5% vs 43.1% (p=0.95)]. Fibrynolysis therapy was not used in the later surveys group [26.7% vs. 0.0% (p=<0.001)] while primary percutaneous coronary intervention (PPCI) became common in the late surveys groups [30.6% vs. 57.8% (p<0.001)], as well as the rate of angiography use and PCI during hospitalization in the late surveys groups [47.9% vs 64.7% (P<0.014)] which reflects the changes in revascularization method over time (Figure 1). Also rate of referrring patients to coronary artery bypass surgery had decliced [11.2% vs 0.9% (P<0.009)]. In addition, the later year surveys group received more aggressive medical therapy, with increased use of DAPT, beta-blockers, ACEi, statins and IIb/IIIa antagonists (Table 3). The need for adjunctive therapy such as IABP, use of vasopressors or need for mechanical ventilation remained similar in both groups. The mean length of stay in the ICCU increased in the late surveys' group, probably reflecting the more intensive therapy in those years' groups, or due to more severe clinical profile of the patients.

**Table 3:** Medication at admission

	Early Surveys (2000-2004)N=108		Late Surveys (2006-2013)N=116		
	N	%	N	%	P
**ASA**	86	79.%6	101	87.1%	0.13
**Clopidogrel**	32	29.9%	67	59.3%	<0.001
**Heparin**	66	61%	89	76.7%	0.011
**LMWH**	31	28.7%	38	32.8%	0.5
**BB**	29	27.1%	73	62.9%	<0.001
**ACEi**	35	32.4%	66	57.4%	<0.001
**Nitrates**	36	33.6%	12	14.5%	0.002
**Statins**	20	19.0%	78	69%	<0.001
**Diuretics**	56	52.3%	70	60.9%	0.199
**Digoxin**	15	13.9%	6	5.3%	0.02
**IIB/IIIA**	17	15.9%	40	34.5%	0.001

### Clinical outcome

**Figure 2:**
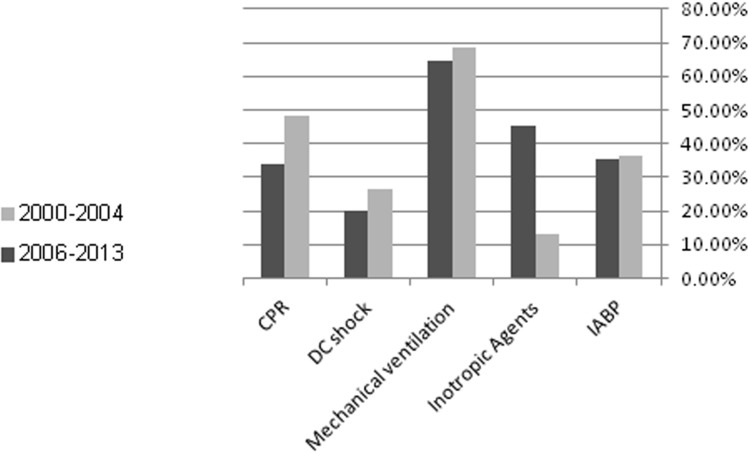
Adjunctive therapy and complication

**Figure 3:**
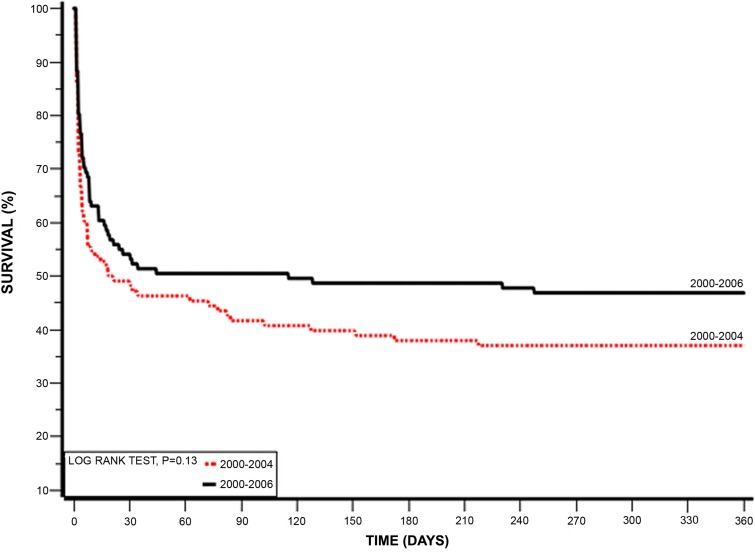
Kaplan-Meier 1 year survival by pre-specified ACSIS early and late time-periods

The later surveys group had a better 7 days mortality rate (44.4% vs. 30.7% (p=0.03)]. This benefit disappeared at 1 year, with similar mortality rate in both groups [63% vs. 53.2% (p=0.14)] and no effect on the rate of 30 days MACE events [(57.4% vs. 47.4% (p=0.13)]. Similar rates of hospital discharges were observed between the two groups (Table 4). During hospital stay similar rates of in hospital mechanical ventilation and electrical complications as well as end organ damage were observed in both groups (Figure 2). The Kaplan-Meier survival analysis showed that at1-year follow-up the cumulative probability of all-cause mortality was the same for both pre-specified early and late time-periods (log-rank P-value for overall mortality during follow up = 0.13) (Figure 3). Multivariate analysis demonstrated that patients from the early survey years compared to those in the late survey years group did not experience a significantly higher risk for 7-days mortality [HR=1.60 95% CI 0.95-2.84 (*p*=0.1)], but the risk for 1-year mortality was significantly higher in the early survey years compared to those in the late survey years group [HR= 1.75 95% CI 1.08-2.81 (p=0.02)]. Further analysis showed that patients admitted with STEMI compared to NSTEMI were independently associated with a significant reduced risk of 1-year mortality [HR=0.54 95% CI 0.30-0.98 (p=0.04)]. Although more patients from the late surveys group were admitted after sudden cardiac death, this appeared not to influence early or late mortality [HR=1.189 95% CI 0.566-2.494 (p=0.6478)] and [HR=0.721 95% CI 0.403-1.289 (=0.269)] respectively. In addition, younger patients (< 75 year old) and those without prior diabetes mellitus admitted with CS had a trend for lower mortality [HR=0.67 95% CI 0.42-1.07 (p=0.09)] and [HR=0.64 95% CI 0.40-1.02 (p=0.06)] respectively.

**Table 4:** Total mortality rates

	2000-2004		2006-2013		
	N=108 (available data)		N=116 (available data)		
	N	%	N	%	P
**Discharge**	44	40.7%	37	31.9%	0.16
**7day mortality**	48	44.4%	35	30.7%	0.03
**30day mortality**	57	52.8%	52	46.4%	0.34
**1year mortality**	68	63.0%	59	53.2%	0.14
**MACE(30 days)**	62	57.4%	55	47.4%	0.13

In order to estimate the effect of PPCI on mortality rates a further subgroup analysis was done by dividing each group of early and late surveys years for those patients who underwent PPCI on admission compared to those who were either treated medically or received late revascularization therapy. Those patients who underwent PPCI had a trend for better survival over 1 year (Table 5).

**Table 5:** Total mortality rates by PPCI during hospital stay

	2000-2004			2006-2013		
	No PPCI on admission	PPCI on admission	P	No PPCI on admission	PPCI on admission	P
	N (=75)	N (=33)		N(=51)	N(65)	
**Discharge**	31(41.3%)	13(39.4%)	0.85	19(37.3%)	18(27.7%)	0.27
**7day mortality**	35(46.7%)	13(39.4%)	0.48	16(31.4%)	19(30.2%)	0.88
**30day mortality**	40(53.3%)	17(51.5%)	0.86	26(53.1%)	26(41.3%)	0.21
**1year mortality**	50(66.7%)	18(54.5%)	0.22	31(63.3%)	28(45.2%)	0.057
**MACE(30 days)**	44(58.7%)	18(54.5%)	0.66	28(54.9%)	27(41,5%)	0.15

**Figure 4:**
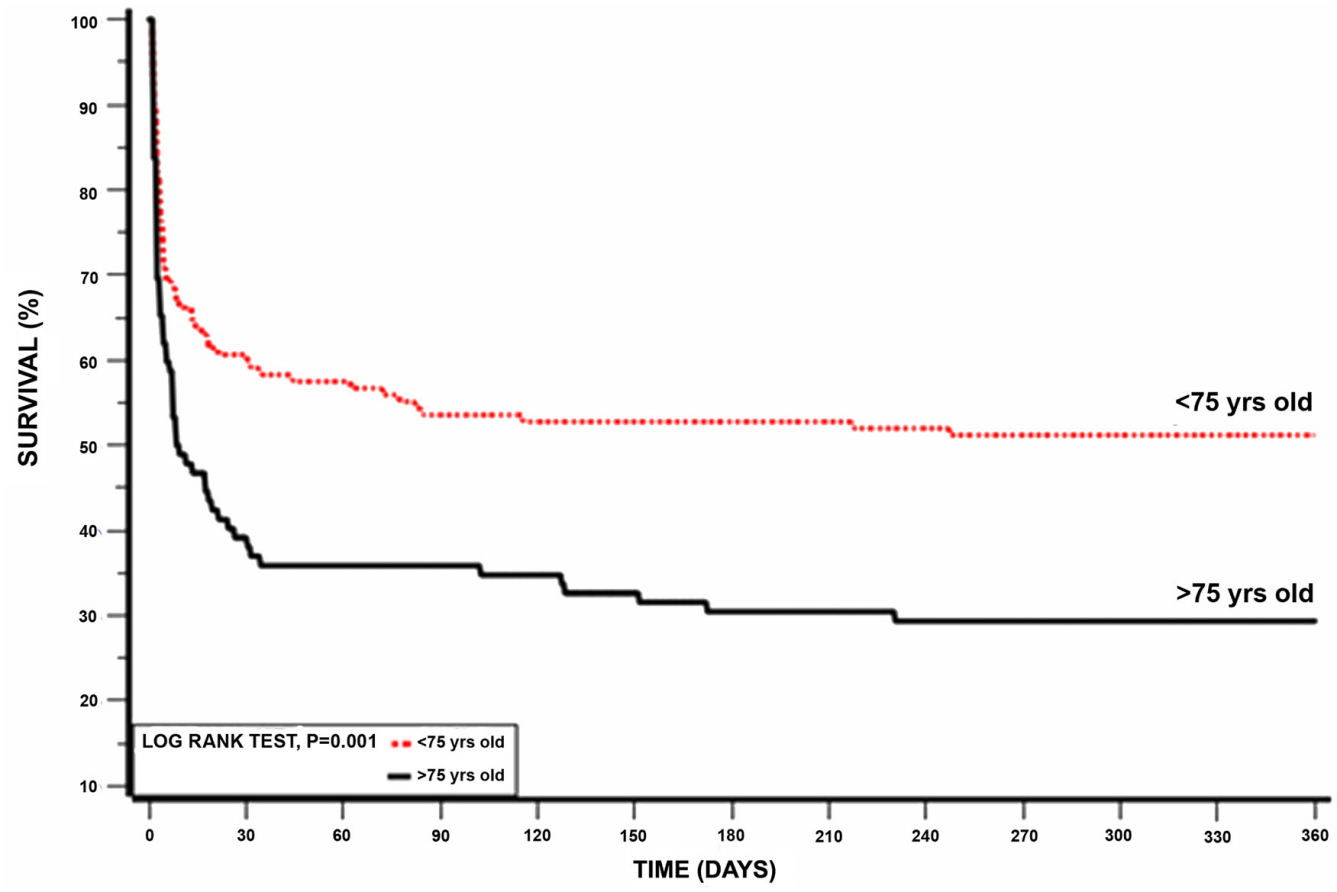
Kaplan-Meier 1 year survival by age (log-rank p = 0.01)

**Figure 5:**
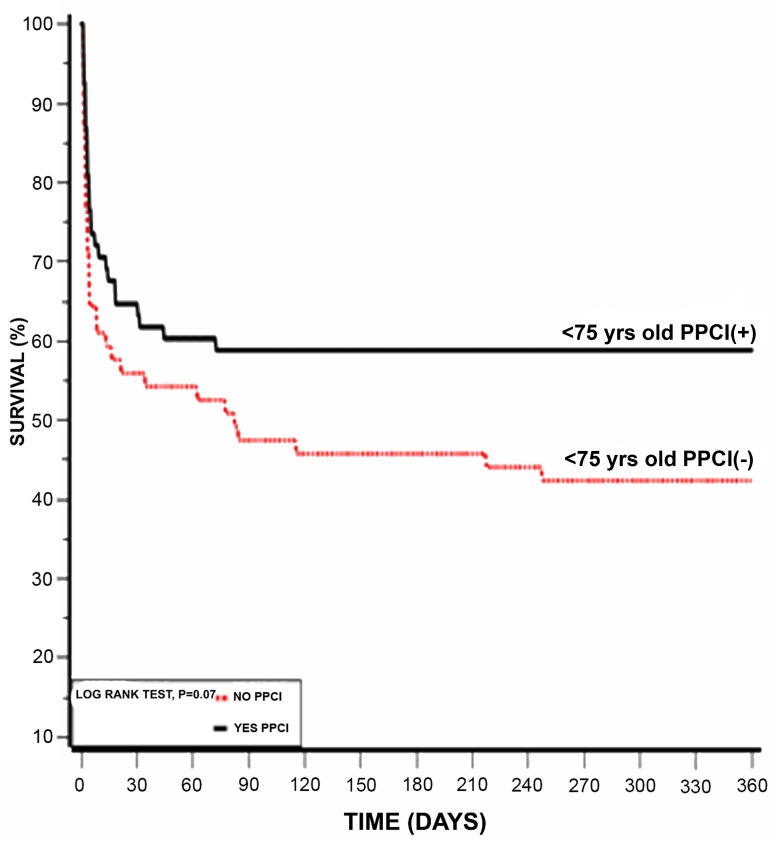

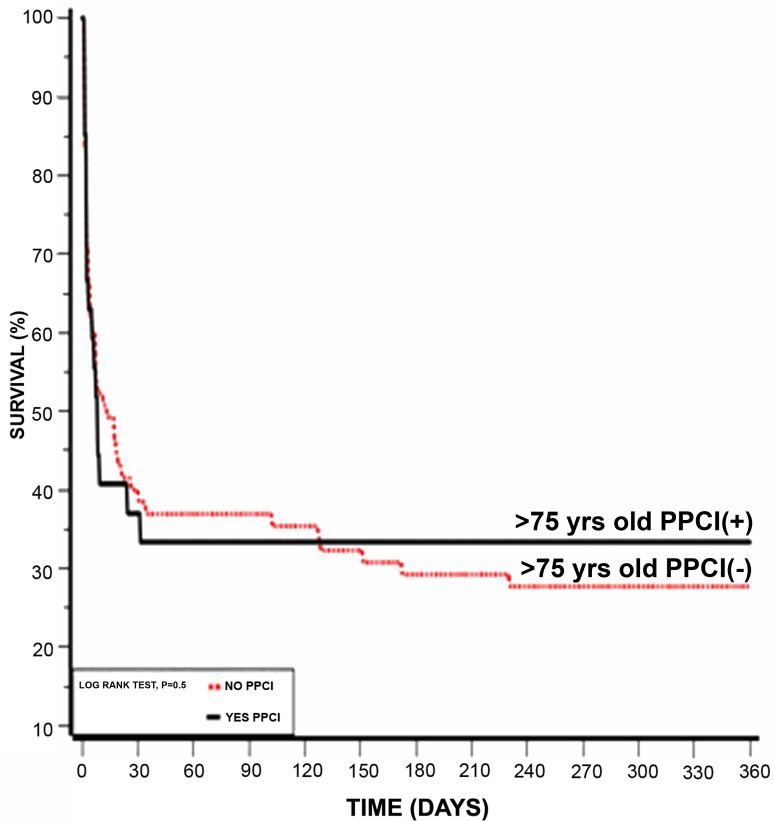
**(a)** Kaplan-Meier 1 year survival of patient underwent PPCI vs. No PPCI on and below 75 years. **(b)** Kaplan-Meier 1 year survival of patient underwent PPCI vs. No PPCI and above 75 years

Another subgroup analysis according to age below and above 75 years old was also done, showing that patients younger than 75 years had better survival rates (Table 6). Kaplan-Meier survival analysis showed that the cumulative probability of 1 year mortality was lower for patients younger than 75 year (log rank p-value for overall mortality during follow up =0.001; Figure 4). To evaluate whether PPCI affects mortality in older patients, we further sub-divided each age group below and above 75 year to those who received PPCI on admission and those who did not. Using Kaplan-Meier survival analysis, patients younger than 75 years old who underwent PPCI kept the advantage for lower one year mortality (log rank p-value =0.001; Figure 5a) while patients older than 75 years had no advantage from immediate revascularization on one year mortality (log rank p-value =0.5; Figure 5b).

**Table 6:** Total mortality by age

	<75 (128)		≥75 (96)		
	N	%	N	%	P
7day mortality	40	31.5%	43	45.3%	0.03
30day mortality	51	40.2%	58	62.4%	P<0. 01
1year mortality	62	48.8%	65	70.7%	P<0. 01
MACE(30 days)	57	44.5%	60	62.5%	P<0. 01

## DISCUSSION

In the current study the subset of patients presented with ACS and complicated with cardiogenic shock was less than 2% of the entire ACS population, which is similar to previous publications, although the annual prevalence is lower. In the later surveys group, higher rates of PPCI, the use of DAPT, heart failure medical treatments were used, reflecting the changes in guidelines recommendations over the years. Even though the mortality rates up to 1 year remained statistically unchanged in both groups, a trend toward a decline of 10% in one-year mortality rate in the later survey groups was observed. The lack of success in reducing the mortality rates over the years could be partly explained by the fact that the later years' surveys patients had more comorbidities, i.e. sicker population than the parallel group. In fact those who presented with CS in this later group were already the most severe and most complicated cases. Reviewing the clinical and the laboratory data of these patients revealed that most cases presented with very high levels of CK and Troponin, indicating the extent of myocardial injury. Most of these patients had already renal failure at presentation reflected by elevated creatinine level, which correlated with the severity of end organ damage and development of systemic inflammatory reaction (SIRS). Furthermore, it was shown that patient presenting with STEMI complicated with CS had lower rates or 1 year mortality compared to NSTEMI. Similar results were reported by Dhaval Kolte et al. and could be explained by underutilizing early invasive therapy and advance treatment or even delay in diagnosis for patients with CS complicating NSTEMI [4]. Our results support that the timing of performing revascularization, i.e., PPCI, did showed a trend in reducing mortality rate.

Similar to the “Should We Emergently Revascularize Occluded Coronaries for Cardiogenic Shock (SHOCK) trial” analysis, in our study younger patients (age below 75 years old) had better survival rates regardless to the intervention, when compared with older patients [7]. Similar results were reported by Dzavik *et al*. who analyzed the SHOCK registry between 1993 and 1997 and reported in-hospital mortality rates of 76% in elderly population [15]. Another large series of CS patients recruited from 1997 to 2006, reported by Jeger *et al*., reported in-hospital death rates of 74% in patients aged 75 years or more [16]. Furthermore early revascularization in patients younger than 75 years old was associated with increased survival rates, while in older patients no significant effect was observed [1–4, 17]. Although our results are consisted with previous reports, a recent analysis of four nationwide surveys in France conducted over a period of 15 years by Nadia Aissaoui et al., have show that mortality declined by 32% in elderly patients. This was explained by higher rates of PPCI that was reported (up to 66.5%) higher than was reported in our cohort [18]. A meta analysis of the 17 retrospective cohort studies (5,323 patients) demonstrated reduced short and intermediate-term mortality in elderly patients with ACS complicated by CS when appropriately-selected for treatment with early revascularization compared to initial medical therapy [19].

All considered, we believe that in these very ill patients, even the most modern and advanced treatment is still a challenging task, and even with the most intensive means of intervention and medications the results remain relatively poor without a significant effect on overall mortality.

## MATERIALS AND METHODS

### Study population

The ACSIS is a national survey conducted in Israel since 1992. Details of these nationwide registries have been previously reported [12–14]. Briefly, ACSIS is conducted during a period of 2 months, once in two years. Data are prospectively collected from all patients admitted and eventually discharged with a diagnosis corresponding to the ACS spectrum in each of the 25 coronary care units and cardiology wards operating in Israel. Demographic and clinical data are recorded on pre-specified forms for all these patients. The discharge diagnoses are recorded as determined by the attending physicians based on clinical, electrocardiographic, and biomarkers criteria. In-hospital and 30-day outcome data are ascertained by hospital chart review, telephone contact, and clinical follow-up data. Patient management is at the discretion of the attending physicians at each center. Mortality data during hospitalization, at 30 days and one year post-hospitalization are determined for all patients from hospital charts and by matching identification numbers of patients with the Israeli National Population Register. All parameters captured by the registry are defined by protocol. The current study population comprises of all patients included in ACSIS between 2000 through 2013 who were admitted with ACS and presented with evidence for cardiogenic shock on admission, defined as by Killip score 4 upon hospital admission. Patients with cardiogenic shock due to mechanical complications were excluded from the current study population.

### Definitions and outcomes

Comparisons and trend calculations were made using data from all 7 surveys by dichotomizing the survey period into 2 pre-specified time-intervals defined as early (surveys 2000 through 2004) and late (surveys 2006 through 2013), reflecting changes in revascularization strategies during this period (i.e. primary percutaneous coronary intervention vs. thrombolysis). Further sub-analysis was done using the data from all 7 surveys. Outcome measures of the present study included the following 7-day, 30-day and 1-year all-cause mortality; and 30-day major adverse cardiac and cerebral events (MACE) defined as recurrent myocardial infarction, recurrent ischemia, stent thrombosis, urgent repeat revascularization, or death.

### Statistical analysis

Characteristics of study participants pre-specified ACSIS early and late time-periods by survey periods were compared using χ*^2^* test for categorical variables and Student's t-test or Wilcoxon rank tests, as appropriate for continuous variables. The Kruskal-Wallis test was used for comparison of non-normally distributed continuous variables. The probability of all-cause mortality during 30-day and 1-year interval was graphically displayed using the Kaplan-Meier method. Cox proportional hazards multivariate- adjusted survival models were used to evaluate the independent effects of treatment groups on 7 days, 30-days and 1-year all-cause mortality results presented as Hazard Ratio (HR) and confidence interval (CI) 95%. Logistic regression was used to evaluate the association between treatment group and MACE, adjusted for gender, age, hypertension, diabetes mellitus, hyperlipidemia, chronic kidney disease, current smoking, coronary artery disease, heart failure, stroke and peripheral vascular disease. Results presented as Odds Ratio (OR) and confidence interval (CI) 95%.

## LIMITATIONS

Although this study included a relatively large number of patients with CS, recruited prospectively during the national surveys over the years, the current data were reviewed and analyzed retrospectively. Furthermore, by sub-dividing into age groups and those with vs. those without PPCI – the groups became smaller. Nevertheless, we believe that these data reflect the true nature of these very sick patients, in whom even advanced revascularization procedures and guidelines-recommended medical therapy have still only modest effect on long-term survival.

## CONCLUSIONS

In spite of these limitations, our real world cohort study results indicate still very high mortality rates of about 50%, in both the early and later survey groups in patients with ACS complicated by CS on admission. Despite the fact that the use of PPCI has increased over the past decade we have not manage to reduce rates of MACE and mortality significantly in this population. The overall treatment of patients presenting with ACS and CS is still challenging, and additional measures may be required to further improve long-term survival.
